# Generative artificial intelligence adoption and the future of the primary care profession: an online survey of UK general practitioners’ experiences and opinions

**DOI:** 10.1136/bmjdh-2026-000253

**Published:** 2026-10-01

**Authors:** Charlotte Blease, Carolina Garcia Sanchez, Jens Gaab, Joseph E Alderman, Luke Allen, Stephanie Kumpunen

**Affiliations:** 1 Department of Women's and Children’s Health, Uppsala Universitet, Uppsala, Uppsala County, Sweden; 2 Division of Digital Psychiatry, Beth Israel Deaconess Medical Center, Harvard Medical School, Boston, Massachusetts, USA; 3 Division of Clinical Psychology and Psychotherapy, University of Basel, Basel, Switzerland; 4 College of Medicine and Health, University of Birmingham, Birmingham, UK; 5 NIHR Birmingham Biomedical Research Centre, Birmingham, UK; 6 University Hospitals Birmingham NHS Foundation Trust, Birmingham, UK; 7 Nuffield Department of Primary Care Health Sciences, University of Oxford, Oxford, UK; 8 Nuffield Trust, London, UK

**Keywords:** Artificial intelligence, Documentation, Primary Health Care, Health Personnel, Medical Informatics

## Abstract

**Objective:**

Use of large language model-based systems in healthcare has expanded rapidly since late 2022. Building on previous national surveys, we examined current generative AI (GenAI) use among UK general practitioners (GPs) and how GPs conceptualise artificial intelligence (AI), its clinical role and implications for professional practice.

**Methods and analysis:**

We analysed data from the 2026 wave of the Generative AI in UK General Practice (GPAI-UK) survey is an annual, convenience sample online survey of UK GPs administered via Doctors.net.uk within a scheduled monthly omnibus targeting approximately 1000 responses. The questionnaire retained core items on GenAI use and added items on conceptualisations of AI, anticipated effects on skills and meaningfulness of work, perceived patient responses, career advice and long-term professional expectations. Descriptive statistics were used to analyse responses.

**Results:**

A total of 1004 GPs completed the online survey. Just over half reported using GenAI in clinical practice (52%, 522/1004), most commonly ChatGPT, Heidi Health and Microsoft Copilot. Among users, 70% (363/522) reported that GenAI had reduced their work burden.

Nearly half conceptualised GenAI as a collaborative technology working alongside clinicians (46%, 462/1004), while around one-fifth viewed it as a simple tool (21%, 209/1004) and 1 in 10 as a potential competitor (10%, 103/1004). Most expected GenAI to play at least a moderate role in routine practice within 5 years (69%, 693/1004).

Looking ahead 10 years, one-third expected GenAI to change which GP skills matter most (34%, 337/1004), and 16% (163/1004) anticipated replacement of some core skills, most often documentation (811/1004), knowledge recall (509/1004) and diagnostic reasoning (442/1004). Many GPs said they would continue to encourage a career in general practice in the context of developments in AI tools; almost half had reservations (48%, 478/1004), nearly, one-third were not able to advise either way (32%, 317/1004). Most expected GenAI to substantially reshape the GP role while leaving GPs central to care (59%, 590/1004).

**Conclusion:**

In this survey, over half of UK GP respondents used GenAI and primarily viewed it as a collaborative technology. GPs anticipated reconfiguration of skills and roles rather than widespread professional displacement. Understanding clinicians’ conceptualisations alongside adoption patterns is important for workforce planning, training and regulation.

**WHAT IS ALREADY KNOWN ON THIS TOPIC**

- Surveys in the UK show increasing use of generative AI (GenAI) tools among general practitioners (GPs) for a range of clinical tasks, but evidence has focused largely on whether clinicians are using these tools rather than how they understand its role or the implications of artificial intelligence for the future of their profession.

**WHAT THIS STUDY ADDS**

- Over half of UK GPs in our survey reported using GenAI, and most conceptualised it as a collaborative technology rather than a simple tool or replacement. GPs anticipated substantial reconfiguration of skills and roles, with mixed expectations about meaningfulness of work, and many report uncertainty about advising future doctors, indicating emerging tensions around professional identity.

**HOW THIS STUDY MIGHT AFFECT RESEARCH, PRACTICE OR POLICY**

- Medical education and postgraduate training should urgently address how GenAI reshapes clinical roles, skills and professional identity. Workforce planning and regulation should attend to clinicians’ conceptual models of AI alongside adoption metrics when considering approaches to integration and professional practice.

## Introduction

Generative AI (GenAI) systems such as ChatGPT, Claude and Copilot have rapidly moved from being experimental curiosities to technologies with visible presence in everyday clinical workflows. Across healthcare, they are increasingly being applied to documentation, clinical reasoning and the delivery of empathic care.^1–3^ In the UK, this uptake is occurring against a backdrop of ambitious national policy: England has committed to becoming the most artificial intelligence (AI)-enabled health system internationally,^4^ making general practice a bellwether for how these technologies reshape frontline clinical work.

Empirical evidence indicates a modest but consistent upward trajectory in adoption. A national survey in early 2024 found that 20% GPs reported using GenAI tools for clinical purposes.^5^ A follow-up survey in 2025 found 25%,^6^ and a UK-wide survey of Royal College of General Practitioners members found 28% using AI tools in their work.^7^ Alongside this growth, concerns persist regarding inaccurate or fabricated outputs, bias, privacy and unclear medicolegal accountability.^7–11^

Most research to date has focused on whether clinicians use GenAI and for which tasks,^5 7 11–13^ with a growing strand examining more disruptive possibilities such as the partial automation of professional work and longer-term task outsourcing.^14–17^ What remains underexamined is how clinicians themselves understand their relationship with these tools.^4^ This matters because the effects of a technology are not fixed in advance: they emerge through the psychological and social contexts of how practitioners interpret it, the routines they build around it and the organisational context in which it sits.^16 18 19^ Whether GenAI becomes a helpful adjunct, a force that reconfigures clinical judgement or a driver of task displacement will depend not only on the tools themselves but on how clinicians and their organisations make sense of them and put them to use.

General practice provides a particularly informative setting in which to examine these questions. General practitioners (GPs) undertake a wide range of cognitive, relational and administrative activities, many of which are directly targeted by current GenAI applications.^5–7^ Their expectations about the future of the profession in an AI-enabled health system can also influence recruitment, retention and training at a time of sustained workforce pressure.^20^ How GPs conceptualise AI—as a tool, collaborator or potential competitor—and how those conceptualisations map onto views about skills, meaningfulness of work and the future of the profession are therefore central to understanding adoption, not peripheral to it.

Building on GPs’ adoption and attitudes towards AI,^5 6 11 21^ the present 2026 survey extends this work by examining how GPs anticipate changes to their skills, professional identity and the profession itself. Given recent policy ambitions around AI (specifically in England),^4^ we added survey items assessing the anticipated effects of GenAI on clinical skills, meaningfulness of work, advice to prospective GPs and views on how general practice may evolve.

### Objectives

This study aimed to examine UK GPs’ use of GenAI in clinical practice alongside their conceptualisations of its role in the profession. Specifically, we sought to: describe current patterns of GenAI use and perceived workload effects; characterise how GPs conceptualise AI in relation to their work; and examine how these conceptualisations relate to expectations about skill change, meaningfulness of work, anticipated patient responses to automation, advice to prospective GPs and views about the future of the profession over the next 5, 10 and 20 years.

## Methods

We analysed the third year—2026 data—from an annual online survey of 1000 UK GPs’ experiences and views regarding GenAI in clinical practice through the Doctors.net.uk platform.^22^ The study is reported in accordance with the Checklist for Reporting Results of Internet E-Surveys (CHERRIES) (online supplemental appendix 1).

10.1136/bmjdh-2026-000253.supp1 Supplementary data

### Survey design

The GPAI-UK-2026 questionnaire comprised 17 questions across four sections, detailed in online supplemental appendix 2. Section A repeated five items previously assessed^5 6^ to enable comparability over time, covering use of GenAI, tasks supported and whether use was perceived to reduce work burdens. Section B was new in 2026 and responsive to emerging issues and growing interest around professional identity and workforce transformation in the next 5, 10, 20 years.^16^ Questions explored GPs’ current perceptions of GenAI as a tool, collaborator or competitor and its anticipated role in routine practice over 5, 10 and 20-year horizons, including effects on traditional clinical skills, professional meaningfulness, patient responses to AI-assisted care and career advice to prospective GPs. Section C consisted of a single optional open-ended question inviting additional comments about AI and general practice (reported in Blease et al Forthcoming). Section D collected sociodemographic and practice characteristics. Most items were single choice. All closed-ended questions were mandatory. The final questionnaire was pre-tested with five UK GPs, with no modifications made, and designed to be completed within approximately 5 min.

10.1136/bmjdh-2026-000253.supp2 Supplementary data

### Survey administration

As in 2024 and 2025,^5 6 11 23^ the study was administered as part of Doctors.net.uk’s monthly omnibus surveys, which aim to secure around 1000 completed questionnaires from among its 272 700 General Medical Council (GMC) registered members.^22^ Registration prevents multiple entries and ensures that potential respondents meeting the survey requirements have access. Sampling was stratified by UK region using GP data from the GMC Data Explorer.^24^

Survey invitations were delivered by email or presented as homepage notifications on Doctors.net.uk according to users’ communication preferences. Participants received a shopping voucher worth £7.50 (US$8.80; €8.83) on completion. Data collection took place from 7 January to 26 January 2026 and closed once 1000 complete GP responses were verified from the 25 500 GPs active on the platform during fieldwork.

In omnibus surveys, respondents are required to answer all closed-ended items to be included in the final sample. The dataset provided to the team by Doctors.net included anonymised aggregate whole numbers and percentages across demographic sub-groups, including sex, age group, role and practice size. Differences were significance tested to 95% by Doctors.net. Individual-level data were not available, nor was it possible to determine whether individual respondents had participated in earlier waves, as participation was anonymous. The study sample was compared with the GMC Registry using χ² tests in Microsoft Excel (online supplemental appendix 3). Full subgroup figures, including non-significant comparisons, are reported in online supplemental appendix 4. In the Results section, we report only those differences that reached statistical significance at the 5% level (p<0.05).

10.1136/bmjdh-2026-000253.supp3 Supplementary data

10.1136/bmjdh-2026-000253.supp4 Supplementary data

## Results

### Respondent characteristics

During the promotion period, 9392 GPs registered on Doctors.net.uk logged in (defined as unique site visitors) and consequently were exposed to the invitation, while 2929 GPs were contacted directly by email. In accordance with CHERRIES, the survey view rate was 11.88% (1116/9392=unique survey visitors/unique site visitors). The participation rate was 94.26% (1,052/1116=unique visitors who agreed to participate/unique first survey page visitors). A total of 1004 GPs completed the survey, resulting in a completion rate of 95.43% (1004/1052=users who finished the survey/users who agreed to participate).

Most respondents were mid-career to late-career clinicians, with 39% (394/1004) aged 46–55 years and 17% (169/1004) aged 56 years or older. Regarding role, most of the sample were GP partners/principals (44%, 438/1004) or salaried GPs (39%, 395/1004), with smaller proportions of locum GPs (13%, 127/1004) and GP registrars (4%, 44/1004). It was not possible to weight the distribution of roles or practice size in the sample against the distribution of roles or practice size at a national level, because these characteristics are not recorded in the GMC’s national GP register and no comparable UK-wide denominator is published (table 1, note). Therefore, the extent to which these results represent the wider GP population cannot be ascertained. Findings relating to role and practice size should be interpreted with caution. The sample was approximately balanced by gender, with 50% (497/1004) of respondents identifying as female, 49% (487/1004) as male, 2% (19/1004) preferring not to say, and one respondent identifying as another gender; women were modestly underrepresented relative to the GP workforce recorded in the GMC register (the population baseline used throughout; online supplemental appendix 3). Respondents were drawn from across all UK countries and regions, with most respondents practising in England (82%). Practice size was skewed towards larger practices: over half (53%) worked in practices with 10 001 or more registered patients, and 36% in practices with 12 501 or more patients. Full sociodemographic and practice characteristics are shown in table 1 and online supplemental appendix 3 presents comparisons with the GMC’s GP register.

**Table 1:** Sociodemographic and practice characteristics of respondents

Category	Characteristic	Number of respondents (all categories total 1004)	Percentage of respondents (all categories total 100%)	GMC registry (n=72 106)
Age	35 or under	99	10%	10%
	36–45	342	34%	38%
	46–55	394	39%	30%
	56 or over	169	17%	22%
Role	GP partner/principal	438	44%	–
	Salaried GP	395	39%	–
	Locum GP	127	13%	–
	GP registrar	44	4%	–
Sex	Male	487	49%	42%
	Female	497	50%	58%
	Other	1	0%	–
	Prefer not to say	19	2%	–
Practice country/region	London	114	11%	15%
	South West England	85	8%	9%
	South East England	125	12%	13%
	West Midlands	91	9%	15%
	East Midlands	75	7%	
	East of England	80	8%	8%
	Yorkshire and Humber	91	9%	12%
	North East England	44	4%	
	North West England	123	12%	11%
	Scotland	98	10%	9%
	Wales	44	4%	4%
	Northern Ireland	34	3%	3%
Practice size (by number of patients)	Up to 5000	109	11%	–
	5001–7500	155	15%	–
	7501–10 000	204	20%	–
	10 001–12 500	170	17%	–
	12 501 or more	366	36%	–

No information is available on role or practice size at a national level in the GMC GP registry.

Source: https://gde.gmc-uk.org/the-register/register-summary/register-data-summary

GMC, General Medical Council; GP, general practitioner.

### UK GPs’ use of GenAI tools

Overall, 52% (522/1004) of respondents reported purposefully using GenAI tools to assist with aspects of clinical practice (table 2), representing a 24 percentage point increase from the 2025 wave. There was significant variation in GenAI use across sociodemographic characteristics. More male than female GPs reported using GenAI, 56% (271/487) compared with 49% (242/497), respectively. GPs aged 35 or under were more likely to use GenAI (67%, 66/99) than all other age groups, and those aged 56 or more were more likely not to use GenAI (64%, 108/169) than all other age groups. Practices with up to 5000 patients were most likely not to use GenAI (68%, 74/109) compared with all other practice sizes. See online supplemental appendix 4 for all subgroup analyses.

**Table 2:** Reported use of GenAI tools and clinical purposes

Category	Characteristic	Number of respondents (n=522)	Percentage of respondents
Tools used	ChatGPT	351	67
	Heidi Health	242	46
	Microsoft Copilot	124	24
	Gemini	71	14
	Claude	18	3
	Med-PaLM	1	0
	Nuance Dragon Ambient eXperience	1	0
	Other (eg, AccuRx Scribe, Anima, Perplexity, Tortus)	73	14
GenAI use cases	Writing letters	268	51
	Real-time documentation during patient consultations (eg, ambient AI tools that listen to the consultation and generate notes automatically)	212	41
	Referrals	161	31
	Documentation after patient consultations (eg, using generative AI tools to draft, summarise or edit clinical notes following the encounter)	159	30
	Differential diagnosis	145	28
	Treatment options	139	27
	Patient summarisation/timelines from prior documentation	65	12
	Medical certification, for example, for employment	18	3
	Other (please specify):	50	10

Base is the 522 respondents who reported purposeful use of GenAI tools in clinical practice. The 'Tools used’ and 'GenAI use cases’ questions were multicoded, with respondents able to select all applicable options. Percentages do not sum to 100.

AI, artificial intelligence; GenAI, generative AI.

Among the 522 respondents reporting GenAI use, the most frequently selected tools were ChatGPT (67%, 351/522), Heidi Health (46%, 242/522) and Microsoft Copilot (24%, 124/522). GPs who reported using GenAI described employing these tools across a broad range of clinical tasks, most often for writing letters (51%, 268/522) and producing real-time documentation during consultations (41%, 212/522) (see table 2).

GP roles in a practice had an influence on use of AI for some tasks. GP partners/principals were significantly more likely to use GenAI to write letters and produce real-time documentation during consultations than locums (56%, 127/226) versus (39%, 26/66) and (49%, 111/226) versus (26%, 17/66), respectively. While locums were significantly more likely to use GenAI for differential diagnosis and treatment options than GP partners/principals (47%, 31/66) vs (21%, 47/226) and (36%, 24/66) versus (21%, 47/226), respectively.

Among GenAI users, 70% (363/522) reported that these tools had, in general, reduced their work burdens. Respondents aged 35 and under (83%, 55/66) were more likely to report reductions in burden compared with all other age groups, and respondents aged 56 or over were more likely to report no reductions (51%, 31/61) compared with all other age groups—mirroring patterns in use reported above.

### GPs’ conceptualisations of the role of AI

When asked how they viewed the current relationship between GPs and GenAI in terms of AI being a tool, collaborator or competitor, almost half (46%, 462/1004) characterised AI as a ‘helpful collaborator working alongside GPs’. 21% (209/1004) described AI as ‘a simple tool (like a stethoscope)’, which was a significantly more common response among certain respondents, including:

- GPs aged 35 or younger compared with GPs aged 56 and over (35%, 35/99) versus (14%, 24/169), respectively.
- GP partners (26%, 112/438) compared with Salaried and Locum GPs (17%; 66/395 and 13%; 17/127, respectively).
- Male GPs rather than female GPs (23%; 114/487 vs 18%; 91/497).

10% (103/1004) viewed AI as ‘a potential competitor capable of replacing some GP tasks’. 23% (230/1004) reported being unsure—with significantly more female GPs reporting being unsure (26%, 127/497) relative to male GPs (19%, 94/487).

Almost one-quarter of respondents reported GenAI would ‘not play a role at all’ or ‘a minor role’ at best (1%, 14/1004) and (22%, 225/1004), respectively. More than one-third of GPs believe AI would play ‘a moderate role’ (37%, 372/1004), and about one-third believed AI would play ‘a major role’ or ‘transformative role’ in their routine clinical practice (20%, 204/1004) 12%, 117/1004), respectively. Locum GPs were significantly more likely to than GP Partners to report believing AI would play a ‘transformative role’ in their clinical practice in the next 5 years (17%, 22/127) versus (9%, 40/438), respectively); 7% (72/1004) were unsure, and both locums and salaried GPs were significantly more unsure than GP partners (10%, 13/127) and (10%, 38/395), respectively, versus (5%, 20/438)).

### GPs’ anticipated effects of AI on skills and meaningfulness in 10 years

Table 3 describes respondents’ views of AI on future skills and meaningfulness in 10 years. One-third expected AI to partly change which GP skills matter most (34%, 337/1004), and 16% (163/1004) anticipated replacement of some core skills. The other half of respondents suggested core skills would remain unchanged (41%; 415/1004) or were unsure (9%; 89/1004). Skills surrounding documentation, medical knowledge recall, diagnostic problem-solving, and referral decision-making were the tasks most frequently identified as likely to be affected by AI.

**Table 3:** Expected effects of AI on GP skills and meaningfulness of work over the next 10 years

Question	Characteristic	Number of respondents (n=1004 or 915; see footnote)	Percentage of respondents (all categories total 100%)
In the next 10 years, do you think AI will reduce reliance on certain traditional GP skills? (select one)	Yes—AI will replace some core skills	163	16
	Partly—AI will change which skills matter most	337	34
	No—core GP skills will remain largely unchanged	415	41
	Unsure	89	9
	Respondent base	1004	100
Over the next 10 years, which GP skills or tasks do you think could be most affected by AI? (select multiple)	Documentation	811	89
	Medical knowledge recall	509	56
	Diagnostic problem-solving	442	48
	Referral decision-making	383	42
	Patient communication	319	35
	Care coordination	291	32
	Empathy	53	6
	Physical examination	37	4
	Other (please specify)	19	2
	Respondent base	915	
Over the next 10 years, what impact—if any—do you expect AI-based tools to have on the meaningfulness of GP work (select one)	Make GP work less meaningful	194	19
	Make GP work more meaningful	225	22
	Have no impact on the meaningfulness of GP work	367	37
	Unsure	218	22
	Respondent base	1004	100
Within the next 10 years, based on your experience with patients, how do you think most patients would respond if AI systems took over some routine GP tasks? (select one)	Very positively	19	2
	Mostly positively	154	15
	Mixed/divided	537	53
	Mostly negatively	171	17
	Very negatively	51	5
	Unsure	72	7
	Respondent base	1004	100

Figures represent the percentage of respondents selecting each option. Sample sizes vary by question: n=1004 for questions answered by all respondents, and n=915 for the question on which skills or tasks AI might affect (asked only of those who expressed a view on to the preceding question on reduced reliance on traditional skills). The ‘Which skills or tasks AI might affect’ question was multicoded, with respondents able to select all applicable options—percentages do not sum to 100. However, for all other questions, respondents could select only one option.

AI, artificial intelligence; GP, general practitioner.

Views on the impact of AI on the meaningfulness of GP work were mixed. Over one-third expected no impact (37%, 367/1004)—which was a more common belief among GPs 35 or under (42%, 42/99) and those aged 46–55 (38%, 148/394) relative to those aged 56 or over (29%, 49/169). 22% (225/1004) anticipated that AI would make work more meaningful (this was more common among males rather than females (28%, 134/487 vs 18%, 89/497, respectively). 19% (194/1004) expected work to become less meaningful—which was a more common belief among Locums rather than GP partners/principals (28%, 35/127 vs 18%, 79/438, respectively) and those aged 56 or more rather than those aged 35 or under (22%, 38/169) vs (12%, 12/99), respectively). The remainder were unsure.

When asked how patients would respond if AI systems took over some routine GP tasks over the next 10 years, 53% (537/1004) anticipated mixed or divided reactions. Smaller proportions expected mostly positive (15%, 154/1004) (though this was more common among male GPs rather than female GPs (20%; 97/487 vs 11%; 57/497, respectively)) or very positive (2%, 19/1004) responses, while 17% (171/1004) anticipated mostly negative and 5% (51/1004) very negative responses. Seven per cent (72/1004) were unsure.

### Career advice and long-term professional futures

Around half of respondents (48%, 478/1004) reported that they would continue to encourage a career in general practice in a context where AI tools continue to develop. This figure included 23% (226/1004) who said they would encourage prospective GPs just as strongly as before AI tools entered the market, and 25% (252/1004) who reported they would encourage some reservations. Smaller proportions reported increased hesitancy (14%, 137/1004) or would discourage a GP career (7%, 72/1004); while nearly one-third did not yet feel able to advise either way (32%, 317/1004).

Looking ahead 20 years, most respondents expected that AI will significantly reshape the GP role in the UK but that GPs will remain central to care (59%, 590/1004). 12% (124/1004) expected the GP role to remain ‘largely unchanged’. Smaller proportions anticipated scenarios in which ‘AI will take over most GP tasks with doctors mainly providing oversight’ (11%, 115/1004) and in which ‘AI will largely replace GPs with minimal doctor involvement’ (3%, 31/1004). 14% (144/1004) were unsure.

## Discussion

### Principal findings

In this online convenience sample survey of 1004 UK GPs, just over half reported having used GenAI tools in clinical practice (52%, 522/1004), and among users, most perceived these tools as reducing work burdens (70%, 363/522). Reported use extended beyond documentation to include administrative and clinical reasoning tasks, indicating that GenAI is already embedded across multiple core domains of general practice.

Nearly half viewed GenAI as a collaborator working alongside clinicians (46%, 462/1004), whereas far fewer described GenAI as a simple tool (21%, 209/1004) or as a potential competitor capable of replacing GP tasks (10%, 103/1004). Although the effectiveness of their use of GenAI was not explored, the predominance of the collaborator frame, rather than the tool or competitor frame, is theoretically significant: it signals that most UK GPs appear to perceive AI as woven into clinical reasoning and workflow rather than sitting alongside it as a discrete instrument. Consistent with this, most respondents expected AI to play at least a moderate role in routine practice within 5 years (69%, 693/1004).

Looking further ahead, 10 years from now, respondents anticipated reconfiguration of professional skills rather than wholesale displacement. One-third expected AI to change which GP skills matter most (34%, 337/1004), while a smaller but notable minority anticipated replacement of some core skills (16%, 163/1004). Documentation, knowledge recall and diagnostic problem-solving were most frequently identified as vulnerable to change.

GPs generally anticipated ambivalent patient responses towards the use of AI in clinical practice, with over half expecting mixed or divided reactions (53%, 537/1004) and more than a fifth (22%, 222/1004) expecting mostly negative or very negative opinions. Workforce implications were prominent: fewer than a quarter (23%, 226/1004) said that would encourage a career in general practice just as strongly as before, and nearly one-third did not yet feel able to advise either way (32%, 317/1004). These findings are consistent with a workforce in the middle of that renegotiation rather than one settled on its outcome.

When considering longer-term futures, most respondents expected AI to significantly reshape the GP role while leaving GPs central to care (59%, 590/1004), whereas only small minorities anticipated that AI would take over most GP tasks (11%, 115/1004) or largely replace GPs (3%, 31/1004). Collectively, these findings indicate that UK GPs view AI as a transformative force for practice and skills. Despite their adoption of GenAI in current practice, and how they conceive of these tools, they did not expect AI to render the profession obsolete. These conceptualisations might be considered in tension.

### Comparison with prior work

Our findings extend the growing UK evidence on GenAI use in general practice. Reported use among UK GPs has risen from 20% in 2024^5^ to 25% in 2025^6^ to 52% in the present survey. Although these are repeated cross-sectional samples rather than a longitudinal cohort, the scale and consistency of the shift across waves suggests recent rapid acceleration rather than incremental diffusion. Other UK evidence points in the same direction.^25^ A 2025 Nuffield Trust survey reported 28% of GPs had used AI tools at work and described widespread informal experimentation occurring in advance of clear organisational governance or national guidance.^7^ Taken together, these UK studies indicate that adoption is advancing more quickly than formal policy, procurement, and training infrastructures, with GPs increasingly integrating GenAI tools—including commercial chatbots—into everyday work. While research in European countries is lacking,^26^ reviews and surveys from the USA describe rapidly expanding clinician AI adoption.^12 13^

Our data also contribute novel insight into how GPs conceptualise AI’s role. Whereas policy discourse often frames AI primarily as a productivity-enhancing tool, nearly half of respondents in our survey conceptualised GenAI as a collaborator. This resonates with emerging theoretical accounts of ‘triadic’ clinical encounters in which patient, clinician and AI jointly shape information production and interpretation.^15 17^ Such framings matter because they may shape how the technology is likely to be used: while it remains to be empirically investigated, practitioners who see AI as a collaborator might be more likely to anthropomorphise it and may be more likely to integrate its outputs into their reasoning.^16^

Patterns across demographic characteristics suggest that GPs’ relationship with AI is shaped by distinct, overlapping influences. As in previous surveys,^6 23^ practice size primarily shaped whether GenAI was used: adoption rose from 32% in the smallest practices (up to 5000 patients) to 58% in the largest (12 501 or more), with documentation-heavy tools such as ambient scribing concentrated in larger organisations—a pattern consistent with the infrastructural and organisational conditions that enable routine tool integration and a significant risk to equity of access to AI tools in general practice.^7^

Again, age shaped adoption in a similar direction^6^ but extended to how AI was experienced and framed: use fell from 67% among GPs aged 35 or under to 36% among those aged 56 or over—this mirrors patterns in another UK-wide survey.^7^ Role, by contrast, shaped what GenAI was used for more than whether it was used: GP partners/principals used it predominantly for administrative tasks (letter-writing, real-time documentation), while locums were more likely to use GenAI for differential diagnosis and treatment options, and were nearly twice as likely to anticipate a transformative role for AI within 5 years. These patterns indicate that the ways GPs encounter, interpret, and use GenAI are shaped not only by individual attitudes but by the infrastructural, biographical and task-related conditions of their work—which suggests the same technology is already becoming different things for differently placed GPs.

UK health policy has placed strong emphasis on digital transformation and AI enablement as part of long-term NHS reform,^4^ but NHS England’s 10-year Health Plan articulates little about implementation including how clinicians and AI should interrelate in everyday practice or how professional roles may evolve. Our respondents, in contrast, express clearer expectations of occupational change than are articulated in current policy.

Indeed, our findings raise specific policy concerns: In England, procurement and use-case guidance for the NHS has concentrated on ambient scribing and the next national use case to be championed is AI triage, which will be a key feature of the NHS App.^27^ Our GP respondents anticipate a broader collaborative role extending into diagnostic reasoning and knowledge recall in the next 10 years. If national procurement and training investment continue to track the narrower, efficiency-focused framing of AI rather than the collaborative role GPs describe, they risk under-serving how genAI is actually being integrated into clinical work. This points to a need for policy, regulation and commissioning to widen the focus beyond documentation tools and plan for genAI’s role in clinical reasoning.

A persistent gap between adoption and preparation is also evident across the UK evidence base: prior survey waves reported minimal employer or commissioner encouragement and almost no formal training despite growing uptake.^6 7 11 21 23^ Even while regulation of AI as a medical device is developing in the UK,^28^ the present findings—particularly uncertainty about career advice and mixed expectations about meaningfulness of work—suggest educational and professional frameworks have not kept pace with technological change. Similar concerns have been raised internationally, with calls for training that addresses not only technical limitations but also professional judgement, accountability and role boundaries.^15 17 29^

GPs’ expectations that patients’ responses to AI will be mixed or divided sit in contrast with evidence that patients are already using GenAI for health information at scale^30–32^ and that a substantial minority of the public (38%) says AI will improve care quality.^33^ This divergence suggests clinicians may be projecting their own uncertainty onto patients, and underscores the need for parallel patient-facing research.^34 35^

The pattern across UK studies is consistent with the emergence of ‘shadow systems’^36^—clinician-driven use of consumer or semi-integrated GenAI tools to manage workload pressures ahead of formal employer endorsement.^7^ Our findings strengthen the case for coordinated national regulation and clinically grounded training and—given the rapid uptake of commercial GenAI tools—for meeting clinicians where adoption already is.

### Strengths and limitations

This study provides one of the earliest large-scale quantitative examinations of how UK GPs conceptualise the clinical role of GenAI and their opinion on the implications of AI for their professional future. A major strength is that this survey forms part of an ongoing annual programme of data collection using a stable core instrument. Although individual respondents cannot be linked across waves, repeated cross-sectional sampling permits examination of secular patterns in adoption and attitudes over time, an approach which is uncommon in research on emerging digital health technologies.

Several limitations should be acknowledged. First, because the survey was administered as part of a scheduled omnibus panel, a conventional response rate could not be calculated. Relatedly, subgroup differences were tested using chi-squared tests rather than multivariable models because Doctors.net supplied the dataset in aggregated, cross-tabulated form rather than as individual-level records. As some subgroups were small, it was not possible to fit multivariable logistic regression models to adjust for potential confounding between demographic characteristics, such as the overlap between age and role. The subgroup differences we report should therefore be read as unadjusted associations rather than independent effects. Second, the regional, gender, and age distributions differed from GMC Registry distributions—and could influence findings. In addition, data on the 5% of respondents (48/1,052) who began but did not complete the survey were not retained by the platform, precluding analysis of whether attrition was associated with particular characteristics or response patterns. Third, participants were recruited from a single professional network using a non-probability sampling approach, which limits robust findings about generalisability and may introduce selection biases toward more digitally engaged GPs. Respondents may also have been disproportionately those with strong views on AI in general practice or with more discretionary time to complete a voluntary survey (eg, part-time GPs or those without external caring responsibilities), while the completion incentive may itself have appealed differentially across these groups. Relatedly, mandatory closed-ended items, while maximising completion, may have prompted satisficing among reluctant respondents rather than genuine engagement. This is a trade-off inherent to forced-choice designs, set against the alternative risk of higher abandonment with optional items.

The survey captured self-reported experiences and views, which cannot be independently verified and may be affected by recall or social desirability bias. In addition, while the online questionnaire provided examples of popular GenAI tools, it did not directly assess respondents’ understanding of AI or level of digital literacy, making it difficult to determine how familiarity with these tools shaped reported perceptions.

Finally, because organisational policies governing authorised use of GenAI in clinical practice remain variable and evolving, some respondents may have been reluctant to disclose use or express candid views. Combined, these limitations should be considered when interpreting the findings.

Future research should move beyond cross-sectional measurement of adoption rates towards closer examination of how GenAI is enacted in everyday general practice. Longitudinal and qualitative methods are particularly suited to tracing how conceptualisations of AI evolve as tools become more embedded, how skill boundaries are renegotiated in situ, and how organisational, educational, and regulatory arrangements shape and are shaped by these enactments. In addition, future research should test whether policy-promoted use cases keep pace with how GPs actually conceptualise and use GenAI. Parallel research with patients is needed to test whether clinicians’ ambivalent expectations of patient response correspond to patient views.

## Conclusion

GenAI is already embedded in UK general practice, often in the guise of consumer tools, including ChatGPT and Microsoft Copilot. Most GPs in our study did not view AI technologies as replacements, but many anticipated substantial changes to skills, roles, and professional identity. Importantly, a notable minority expressed uncertainty about the future of the GP role or anticipate task displacement, indicating emerging professional unease alongside more optimistic expectations.

Policy now clearly lags behind practice: while GPs increasingly report active adoption of GenAI in their everyday work, national frameworks remain vague about how doctors and AI should coexist in healthcare. Without urgent attention to training, professional boundaries, and governance, GenAI risks becoming normalised in primary care by default rather than by design.

## Data Availability

Data are available on reasonable request.
