# Supplementary material for: Generative artificial intelligence adoption and the future of the primary care profession: an online survey of UK general practitioners’ experiences and opinions
Source: BMJ Digit Health Ai. 2026 Oct 1;2(1):e000253. doi: 10.1136/bmjdh-2026-000253 (PMC13663762; doi:10.1136/bmjdh-2026-000253)
Supplement: Supplementary file 1 [file bmjdh-2-1-s001.pdf]

Appendix 1. Checklist for Reporting Results of Internet E-Surveys (CHERRIES)

Item Category	Checklist item	Described in the manuscript	Cited from the manuscript
**Design**	Describe survey design	Yes	In **Methods > Survey Design p.7-8**
**IRB (Institutional Review Board) approval and informed consent process**	IRB approval	Yes	**Methods > Ethical considerations** As UK GPs were recruited via a third-party commercial platform in their professional capacity, rather than as NHS patients or via NHS data or premises, UK Health Research Authority approval was not required. Ethical approval was granted by the Faculty of Psychology, University of Basel (approval number 032-25-1). **p.9**
	Informed consent	Yes	**Methods** > **Ethical considerations** Participation is voluntary and respondents provided informed consent before taking part. **p.9**
	Data protection	Yes	**Methods** > **Ethical considerations** The survey was administered through Doctors.net.uk, a secure platform that uses encryption and anonymisation to prevent linkage between responses and personal identifiers. All identifiable information, including email addresses, was removed prior to transfer of the dataset to the research team. Doctors.net.uk complies with General Data Protection Regulation. **p.9**
**Development and pre-testing**	Development and testing	Yes	**Methods > Survey Design** The final questionnaire was pre-tested with five UK GPs, with no modifications made, and designed to be completed within approximately five minutes. **p.7**
**Recruitment process and description of the sample having access to the questionnaire**	Open survey versus closed survey	Yes	**Methods > Survey Administration** As in 2024 and 2025 [5,6,11,23], the study was administered as part of Doctors.net.uk's monthly omnibus surveys, which aim to secure around 1000 completed questionnaires from among its 272,700 General Medical Council (GMC) registered members [22]. **p.8**

	Contact mode	Yes	**Methods > Survey Administration**<br>Survey invitations were delivered by email or presented as homepage notifications on Doctors.net.uk according to users' communication preferences. **p.8**
	Advertising the survey	Yes	**Methods > Survey Administration**<br>Survey invitations were delivered by email or presented as homepage notifications on Doctors.net.uk according to users' communication preferences. **p.8**
**Survey administration**	Web/E-mail	Yes	**Methods > Survey Administration**<br>Survey invitations were delivered by email or presented as homepage notifications on Doctors.net.uk according to users' communication preferences. **p.8**
	Context	Yes	**Methods > Survey Administration**<br>As in 2024 and 2025 [5,6,11,23], the study was administered as part of Doctors.net.uk's monthly omnibus surveys, which aim to secure around 1000 completed questionnaires from among its 272,700 General Medical Council (GMC) registered members [22]. **p.8**
	Mandatory/voluntary	Yes	**Methods > Ethical considerations**<br>Participation is voluntary and respondents provided informed consent before taking part. **p.9**
	Incentives	Yes	**Methods > Survey Administration**<br>Participants received a shopping voucher worth £7.50 (US$8.80; €8.83) upon completion. **p.8**
	Time/Date	Yes	**Methods > Survey Administration**<br>Data collection took place from 7 January to 26 January 2026 and closed once 1000 complete GP responses were verified from the 25,500 GPs active on the platform during fieldwork. **p.8**
	Randomization of items or questionnaires	No	**--**
	Adaptive questioning	No	**--**
	Number of Items	No	**--**
	Number of screens (pages)	No	**--**
	Completeness check	Yes	**Methods > Survey Administration**<br>In omnibus surveys, respondents are required to answer all closed-ended items to be included in the final sample. **p.8**

	Review step	No	--
**Response rates**	Unique site visitor	Yes	**Results > Respondent Characteristics**<br>During the promotion period 9,392 GPs registered on Doctors.net.uk logged in (defined as unique site visitors) and consequently were exposed to the invitation, while 2,929 GPs were contacted directly by email. **p.9**
	View rate (Ratio of unique survey visitors/unique site visitors)	Yes	**Results > Respondent Characteristics**<br>In accordance with CHERRIES the survey view rate was 11.88% (1,116 /9,392 = unique survey visitors / unique site visitors). **p.9**
	Participation rate (Ratio of unique visitors who agreed to participate/unique first survey page visitors)	Yes	**Results > Respondent Characteristics**<br>The participation rate was 94.26% (1,052 /1,116 = unique visitors who agreed to participate / unique first survey page visitors). **p.9**
	Completion rate (Ratio of users who finished the survey/users who agreed to participate)	Yes	**Results > Respondent Characteristics**<br>A total of 1004 GPs completed the survey, resulting in a completion rate of 95.43% (1,004 /1,052 = users who finished the survey / users who agreed to participate). **p.9**
**Preventing multiple entries from the same individual**	Cookies used	No	Not used as a means to prevent multiple entries.
	IP check	No	Not used as a means to prevent multiple entries.
	Log file analysis	No	Not used as a means to prevent multiple entries.
	Registration	No	**Methods > Survey Administration**<br>Registration prevents multiple entries and ensures that potential respondents meeting the survey requirements have access. **p.8**
**Analysis**	Handling of incomplete questionnaires	Yes	**Methods > Survey Administration**<br>As in 2024 and 2025 [5,6,11,23], the study was administered as part of Doctors.net.uk's monthly omnibus surveys, which aim to secure around 1000 completed questionnaires from among its 272,700 General Medical Council (GMC) registered members [22]. […]<br>In omnibus surveys, respondents are required to answer all closed-ended items to be included in the final sample. **p.8**
	Questionnaires submitted with an atypical timestamp	No	--
	Statistical correction	No	--

Reference:

Eysenbach G. Improving the quality of Web surveys: the Checklist for Reporting Results of Internet E-Surveys (CHERRIES). J Med Internet Res 2004;6:e34.
