# Supplementary material for: Generative artificial intelligence adoption and the future of the primary care profession: an online survey of UK general practitioners’ experiences and opinions
Source: BMJ Digit Health Ai. 2026 Oct 1;2(1):e000253. doi: 10.1136/bmjdh-2026-000253 (PMC13663762; doi:10.1136/bmjdh-2026-000253)
Supplement: Supplementary file 2 [file bmjdh-2-1-s002.pdf]

Appendix 2. Survey Questions

Q1a [Single response permitted. Mandatory.]

Have you ever purposely used any generative AI tools (e.g. ChatGPT, Med-PaLM) including ambient AI scribes (e.g. Heidi Health), to assist you in any aspect of clinical practice?

- Yes
- No

If yes to Q1a: Q1b [Selection of multiple options permitted. No limit to number of options selected. Mandatory.]

What tools have you used?

- ChatGPT
- Heidi Health
- Microsoft Copilot
- Gemini
- Claude
- Med-PaLM
- Nuance Dragon Ambient eXperience (DAX)
- Other (please specify)

If yes to Q1b: Q1c [Selection of multiple options permitted. No limit to number of options selected. Mandatory.]

For what purpose(s) have you used generative AI tools?

- Writing letters
- Real-time documentation during patient consultations (e.g. ambient AI tools that listen to the consultation and generate notes automatically)
- Referrals
- Documentation after patient consultations (e.g. using generative AI tools to draft, summarise, or edit clinical notes following the encounter)
- Differential diagnosis
- Treatment options
- Patient summarization/timelines from prior documentation
- Medical certification e.g., for employment
- Other (please specify):

If yes to Q1b: Q1d [Single response permitted. Mandatory.]

In general, have these tools reduced your work burdens?

- Yes
- No

Q2 [Single response permitted. Mandatory.]

How do you view the relationship between GPs and AI in healthcare?

- A simple tool (like a stethoscope)
- A helpful collaborator (AI + GP working as a team)
- A potential competitor that could replace GP tasks
- Unsure

Q3 [Single response permitted. Mandatory.]

Within the next 5 years, how significant a role do you expect AI to play in your routine clinical practice?

- Not at all
- Minor role
- Moderate role
- Major role
- Transformative
- Unsure

Q4av [Single response permitted. Mandatory.]

In the next 10 years, do you think AI will reduce reliance on certain traditional GP skills?

- Yes - AI will replace some core skills
- Partly - AI will change which skills matter most
- No - core GP skills will remain largely unchanged
- Unsure

Q4b [Selection of multiple options permitted. No limit to number of options selected. Mandatory.]

Over the next 10 years, which GP skills or tasks do you think could be most affected by AI?

- Documentation
- Medical knowledge recall
- Diagnostic problem-solving
- Referral decision-making
- Patient communication
- Care coordination
- Empathy
- Physical examination
- Other (please specify)

Q5 [Single response permitted. Mandatory.]

Over the next 10 years, what impact - if any - do you expect AI-based tools to have on the meaningfulness of GP work

- Make GP work less meaningful
- Make GP work more meaningful
- Have no impact on the meaningfulness of GP work
- Unsure

Q6 [Single response permitted. Mandatory.]

Within the next 10 years, based on your experience with patients, how do you think most patients would respond if AI systems took over some routine GP tasks?

- Very positively
- Mostly positively
- Mixed / divided
- Mostly negatively
- Very negatively
- Unsure

Q7 [Single response permitted. Mandatory.]

If someone were considering a career as a GP, how - if at all - would developments in AI tools affect your advice to them?

- I would encourage them just as strongly as before
- I would encourage them, but with reservations
- I would be more hesitant than before
- I would discourage them
- I don’t yet feel able to advise either way

Q8 [Single response permitted. Mandatory.]

Looking ahead 20 years, which of the following best reflects how you expect the GP profession in the UK to change?

- The GP role will remain largely unchanged
- AI will significantly reshape the GP role, but GPs will remain central to care
- AI will take over most GP tasks, with doctors mainly providing oversight
- AI will largely replace GPs, with minimal doctor involvement
- Unsure

Q9 [Free text. No word limit.]

Do you have any further comments about AI and general practice?

- *Open-ended optional free-text response*

Sociodemographic and Practice Characteristics

S1 [Single response permitted. Mandatory.]

Which of the following best describes your role?

- GP Partner / Principal
- Salaried GP
- Locum GP
- GP Registrar

S2 [Single response permitted. Mandatory.]

Where are you currently practicing?

- London
- South West
- South East
- West Midlands
- East Midlands
- East of England
- Yorkshire and Humber
- North East
- North West

- Scotland
- Wales
- Northern Ireland

D1 [Single response permitted. Mandatory.]

Are you...

- Male
- Female
- Other
- Prefer not to say

D2 [Single response permitted. Mandatory.]

Are you...

- 35 or under
- 36 to 45
- 46 to 55
- 56 or over

D3 [Single response permitted. Mandatory.]

Which of the following best describes the area where your practice is based?

- NHS North Central London ICB
- NHS North East London ICB
- NHS North West London ICB
- NHS South East London ICB
- NHS South West London ICB
- NHS Bath and North East Somerset, Swindon and Wiltshire ICB
- NHS Bristol, North Somerset and South Gloucestershire ICB
- NHS Cornwall and the Isles of Scilly ICB
- NHS Devon ICB
- NHS Dorset ICB
- NHS Gloucestershire ICB
- NHS Somerset ICB
- NHS Bedfordshire, Luton and Milton Keynes ICB
- NHS Buckinghamshire, Oxfordshire and Berkshire West ICB
- NHS Frimley ICB
- NHS Hampshire and Isle of Wight ICB
- NHS Kent and Medway ICB
- NHS Surrey Heartlands ICB

- NHS Sussex ICB
- NHS Birmingham and Solihull ICB
- NHS Black Country ICB
- NHS Coventry and Warwickshire ICB
- NHS Herefordshire and Worcestershire ICB
- NHS Shropshire, Telford and Wrekin ICB
- NHS Staffordshire and Stoke-on-Trent ICB
- NHS Derby and Derbyshire ICB
- NHS Leicester, Leicestershire and Rutland ICB
- NHS Lincolnshire ICB
- NHS Northamptonshire ICB
- NHS Nottingham and Nottinghamshire ICB
- NHS Cambridgeshire and Peterborough ICB
- NHS Hertfordshire and West Essex ICB
- NHS Mid and South Essex ICB
- NHS Norfolk and Waveney ICB
- NHS Suffolk and North East Essex ICB
- NHS South Yorkshire ICB
- NHS Humber and North Yorkshire ICB
- NHS West Yorkshire ICB
- NHS North East and North Cumbria ICB
- NHS Cheshire and Merseyside ICB
- NHS Greater Manchester ICB
- NHS Lancashire and South Cumbria ICB
- NHS Ayrshire and Arran
- NHS Borders
- NHS Dumfries and Galloway
- NHS Fife
- NHS Forth Valley
- NHS Grampian
- NHS Greater Glasgow and Clyde
- NHS Highland
- NHS Lanarkshire
- NHS Lothian
- NHS Shetland
- NHS Tayside
- NHS Western Isles (Bòrd SSN nan Eilean Siar)
- Aneurin Bevan University Health Board
- Betsi Cadwaladr University Health Board
- Cardiff and Vale University Health Board
- Cwm Taf Morgannwg University Local Health Board
- Hywel Dda University Health Board
- Powys Teaching Health Board
- Swansea Bay University Local Health Board
- Belfast Health and Social Care Trust
- Northern Health and Social Care Trust
- South Eastern Health and Social Care Trust
- Southern Health and Social Care Trust
- Western Health and Social Care Trust
- Other

D4 [Single response permitted. Mandatory.]

How many patients do you have on your practice list?

- Up to 5,000 patients
- 5,001–7,500 patients
- 7,501–10,000 patients
- 10,001–12,500 patients
- 12,501 patients or more
