# Supplementary material for: Generative artificial intelligence adoption and the future of the primary care profession: an online survey of UK general practitioners’ experiences and opinions
Source: BMJ Digit Health Ai. 2026 Oct 1;2(1):e000253. doi: 10.1136/bmjdh-2026-000253 (PMC13663762; doi:10.1136/bmjdh-2026-000253)
Supplement: Supplementary file 3 [file bmjdh-2-1-s003.pdf]

Appendix 3. Comparison of Study Sample with the General Medical Council (GMC) Registry

Table 1. Gender

	**Sample** (n = 984)	**GMC Registry** (n = 72,106)	Chi-square Analysis
			χ^2 (1) = 22.64, $p < 0.001$, Cramer's V = 0.15
**Female**	497 (50%)	41,867 (58%)	
**Male**	487 (49%)	30,239 (42%)	

Note: 19 (2%) participants responded 'Prefer not to say' and 1 (0%) participants responded 'Other' and their responses were excluded from the analysis. GMC Registry data included GPs with a licence who are current GPs, including trainees.
Source: *https://gde.gmc-uk.org/the-register/register-summary/register-data-summary*

Table 2. Age range.

	**Sample** (n = 1,004)	**GMC Registry** (n = 72,106)
**35 years or younger**	99 (10%)	7,190 (10%)
**36 – 45 years**	342 (34%)	27,164 (38%)
**46 – 55 years**	394 (39%)	21,810 (30%)
**56 years or older**	169 (17%)	15,942 (22%)

Note: Age ranges in the GMC Registry do not match the ones in the survey, so a statistical comparison was not carried out. GMC Registry ranges are 25-34, 35-44, 45-54, 55+.
Source: *https://gde.gmc-uk.org/the-register/register-summary/register-data-summary*

Table 3. Location.

	**Sample** (n = 1,004)	**GMC Registry** (n = 71,725)	Chi-square Analysis
			χ^2 (9) = 18.76, p < 0.05, Cramer's V = 0.046
**England**			
North West	123 (12%)	7,671 (11%)	
North East and Yorkshire	135 (13%)	8,666 (12%)	
Midlands	166 (17%)	10,431 (15%)	
East of England	80 (8%)	6,050 (8%)	
London	114 (11%)	10,884 (15%)	
South East	125 (12%)	9,423 (13%)	
South West	85 (8%)	6,748 (9%)	
**Northern Ireland**	34 (3%)	2,096 (3%)	
**Scotland**	98 (10%)	6,672 (9%)	
**Wales**	44 (4%)	3,084 (4%)	

Note: The data includes all current GPs, including those who are specialists and trainees, but excludes those who selected their region as 'other'.
Source: *https://gde.gmc-uk.org/the-register/register-by-uk-map/licensed-doctors-by-nhs-region*

Blease C, *et al. BMJ Digit Health* 2026; 2:e000253. doi: 10.1136/bmjdh-2026-000253

Table 4. Comparison of sociodemographic characteristics of GenAI users and non-users GPs.

	**Users** (n = 513 and n = 522)	**Non-users** (n = 482)	Chi-square Analysis
**Gender***			χ^2 (1) = 4.49, p <0.05, Cramer's V = 0.068
Woman	242 (49%)	255 (51%)	
Man	271 (56%)	216 (44%)	
**Age**			χ^2 (3) = 26.61, p <0.001, Cramer's V = 0.163
35 years or under	66 (67%)	33 (33%)	
36 – 45 years	186 (54%)	156 (46%)	
46 – 55 years	209 (53%)	185 (47%)	
56 years or over	61 (36%)	108 (64%)	
**Practice Size**			χ^2 (4) =24.45, p <0.001, Cramer's V = 0.156
Up to 5,000 patients	35 (32%)	74 (68%)	
5,001-7,500 patients	85 (55%)	70 (45%)	
7,501-10,000 patients	99 (49%)	105 (51%)	
10,001-12,500 patients	90 (53%)	80 (47%)	
12,501 patients or more	213 (58%)	153 (42%)	

Note: All analyses are based on the whole sample (*n* = 1,004), except for *Gender* (*n* = 984), where responses *'Prefer not to say'* (*n* = 20) were omitted.
