# Supplementary material for: Generative artificial intelligence adoption and the future of the primary care profession: an online survey of UK general practitioners’ experiences and opinions
Source: BMJ Digit Health Ai. 2026 Oct 1;2(1):e000253. doi: 10.1136/bmjdh-2026-000253 (PMC13663762; doi:10.1136/bmjdh-2026-000253)
Supplement: Supplementary file 4 [file bmjdh-2-1-s004.pdf]

Appendix 4. Data tables including sub-group analyses

Table 1. Respondents' purposeful use of GenAI tools in clinical practice, by gender, age group, role and practice size (% and number)

	**Total**	**Gender**		**Age group**				**Role**			**Practice size by patient number**				
	Total (n=1,004)	Male (n=487)	Female (n=497)	35 or under (n=99)	36 to 45 (n=342)	46 to 55 (n=394)	56 or over (n=169)	GP Partner/ Principal (n=438)	Salaried GP (n=395)	Locum GP (n=127)	Up to 5,000 (n=109)	5,001-7,500 (n=155)	7,501-10,000 (n=204)	10,001-12,500 (n=170)	12,501 or more (n=366)
	A	B	C	D	E	F	G	H	I	J	K	L	M	N	O
Yes	52% (522)	56%[C] (271)	49% (242)	67%[E,F,G] (66)	54%[G] (186)	53%[G] (209)	36% (61)	52% (226)	49% (195)	52% (66)	32% (35)	55% (85)	49% (99)	53% (90)	58% (213)
No	48% (482)	44% (216)	51%[B] (255)	33% (33)	46%[D] (156)	47%[D] (185)	64%[D,E,F] (108)	48% (212)	51% (200)	48% (61)	68%[LMNO] (74)	45% (70)	51%[O] (105)	47% (80)	42% (153)

Note: Single response permitted. Mandatory question. Percentages may not sum to 100 due to rounding. Statistically significant differences are calculated at a 95% confidence level and indicated by column letters. For example, male GPs were significantly more likely to report use than female GPs (56% vs 49%). AI tool use varied markedly by age: GPs aged 35 or under were significantly more likely to report use than all three older age groups (67% vs 54%, 53%, and 36% respectively). GPs aged 56 or over were significantly less likely to report use than all younger age groups.

Table 2. GenAI tools used, by respondents' gender, age group, role and practice size (% and number)

	**Total**	**Gender**		**Age group**				**Role**			**Practice size by patient number**				
	Total (n=522)	Male (n=271)	Female (n=242)	35 or under (n=66)	36 to 45 (n=186)	46 to 55 (n=209)	56 or over (n=61)	GP Partner/ Principal (n=226)	Salaried GP (n=195)	Locum GP (n=66)	Up to 5,000 (n=35)	5,001-7,500 (n=85)	7,501-10,000 (n=99)	10,001-12,500 (n=90)	12,501 or more (n=213)
	A	B	C	D	E	F	G	H	I	J	K	L	M	N	O
ChatGPT	67% (351)	70% (189)	65% (158)	76% (50)	67% (124)	64% (133)	72% (44)	66% (150)	62% (121)	76%[I] (50)	69% (24)	75%[O] (64)	67% (66)	72% (65)	62% (132)
Heidi Health	46% (242)	47% (128)	45% (110)	42% (28)	51%[G] (95)	49%[G] (102)	28% (17)	50%[J] (114)	49%[J] (95)	35% (23)	29% (10)	44% (37)	46% (46)	50%[K] (45)	49%[K] (104)
Microsoft Copilot	24% (124)	32%[C] (86)	15% (37)	17% (11)	23% (43)	27% (57)	21% (13)	27% (60)	19% (38)	23% (15)	37%[N] (13)	28% (24)	24% (24)	19% (17)	22% (46)
Gemini	14% (71)	20%[C] (55)	6% (15)	11% (7)	16% (29)	13% (28)	11% (7)	12% (26)	14% (27)	15% (10)	20% (7)	13% (11)	12% (12)	11% (10)	15% (31)
Claude	3% (18)	7%[C] (18)	0% (0)	0% (0)	6%[D] (11)	2%[D] (5)	3% (2)	4% (8)	5% (9)	2% (1)	3% (1)	5% (4)	4% (4)	2% (2)	3% (7)
Med-PaLM	0% (1)	0% (1)	0% (0)	0% (0)	0% (0)	0% (0)	2% (1)	0% (1)	0% (0)	0% (0)	0% (0)	0% (0)	0% (0)	0% (0)	0% (1)
Nuance Dragon Ambient eXperience (DAX)	0% (1)	0% (1)	0% (0)	0% (0)	0% (0)	0% (1)	0% (0)	0% (1)	0% (0)	0% (0)	0% (0)	0% (0)	0% (0)	0% (0)	0% (1)

Other (please specify)	14% (73)	14% (37)	14% (34)	6% (4)	13% (25)	17%[D] (36)	13% (8)	15% (35)	15% (29)	11% (7)	14% (5)	9% (8)	13% (13)	12% (11)	17% (36)

Note: Selection of multiple options permitted. No limit to number of options selected. Mandatory. Statistically significant differences are calculated at a 95% confidence level and indicated by column letters.

Table 3. Purposes of use of GenAI tools, by respondents' gender, age group, role and practice size (% and number)

	Total	Gender		Age group				Role			Practice size by patient number				
	Total (n=522)	Male (n=271)	Female (n=242)	35 or under (n=66)	36 to 45 (n=186)	46 to 55 (n=209)	56 or over (n=61)	GP Partner/ Principal (n=226)	Salaried GP (n=195)	Locum GP (n=66)	Up to 5,000 (n=35)	5,001-7,500 (n=85)	7,501-10,000 (n=99)	10,001-12,500 (n=90)	12,501 or more (n=213)
	A	B	C	D	E	F	G	H	I	J	K	L	M	N	O
Writing letters	51% (268)	49% (132)	55% (134)	47% (31)	55%[G] (102)	54%[G] (113)	36% (22)	56%[J] (127)	48% (94)	39% (26)	34% (12)	55%[K] (47)	46% (46)	62%[K,M] (56)	50% (107)
Real-time documentation during patient consultations (e.g. ambient AI tools that listen to the consultation and generate notes automatically)	41% (212)	44% (118)	38% (92)	33% (22)	43%[G] (80)	44%[G] (92)	30% (18)	49%[I,J] (111)	39%[J] (76)	26% (17)	26% (9)	33% (28)	40% (40)	40% (36)	46%[K,L] (99)
Referrals	31% (161)	32% (87)	30% (73)	26% (17)	38%[F,G] (71)	29% (60)	21% (13)	33% (74)	31% (60)	23% (15)	23% (8)	34% (29)	29% (29)	29% (26)	32% (69)
Documentation after patient consultations (e.g. using generative AI tools to draft, summarise, or edit clinical notes following the encounter)	30% (159)	31% (85)	30% (72)	35% (23)	32%[G] (59)	31% (65)	20% (12)	29% (65)	34% (67)	26% (17)	23% (8)	27% (23)	31% (31)	40% (36)	29% (61)
Differential diagnosis	28% (145)	32%[C] (87)	23% (55)	32% (21)	27% (51)	22% (47)	43%[E,F] (26)	21% (47)	27% (53)	47%[H,I] (31)	40%[M] (14)	29% (25)	18% (18)	32%[M] (29)	28% (59)
Treatment options	27% (139)	33%[C] (89)	19% (47)	35%[F] (23)	25% (46)	20% (42)	46%[E,F] (28)	21% (47)	28% (55)	36%[H] (24)	40% (14)	26% (22)	25% (25)	28% (25)	25% (53)
Patient summarization/timelines from prior documentation	12% (65)	17%[C] (47)	7% (17)	14% (9)	13% (25)	12% (25)	10% (6)	13% (29)	12% (24)	9% (6)	9% (3)	14% (12)	12% (12)	13% (12)	12% (26)
Medical certification e.g., for employment	3% (18)	4% (10)	3% (8)	5% (3)	3% (6)	4% (8)	2% (1)	3% (7)	4% (8)	3% (2)	0% (0)	2% (2)	5%[K] (5)	2% (2)	4%[K] (9)
Other (please specify)	10% (50)	8% (21)	11% (27)	3% (2)	8% (14)	13%[D] (27)	11% (7)	11% (24)	11% (21)	8% (5)	9% (3)	14% (12)	11% (11)	8% (7)	8% (17)

Note: Selection of multiple options permitted. No limit to number of options selected. Mandatory. Statistically significant differences are calculated at a 95% confidence level and indicated by column letters.

Table 4. Perceptions of current reduced work burdens due to GenAI, by respondents' gender, age group, role and practice size (% and number)

	Total	Gender		Age group				Role			Practice size by patient number				
	Total (n=522)	Male (n=271)	Female (n=242)	35 or under (n=66)	36 to 45 (n=186)	46 to 55 (n=209)	56 or over (n=61)	GP Partner/ Principal (n=226)	Salaried GP (n=195)	Locum GP (n=66)	Up to 5,000 (n=35)	5,001-7,500 (n=85)	7,501-10,000 (n=99)	10,001-12,500 (n=90)	12,501 or more (n=213)
	A	B	C	D	E	F	G	H	I	J	K	L	M	N	O
Yes	70% (363)	71% (192)	68% (165)	83%[E,F,G] (55)	70%[G] (131)	70%[G] (147)	49% (30)	72% (162)	67% (130)	61% (40)	63% (22)	78%[O] (66)	70% (69)	71% (64)	67% (142)
No	30% (159)	29% (79)	32% (77)	17% (11)	30%[D] (55)	30%[D] (62)	51%[D,E,F] (31)	28% (64)	33% (65)	39% (26)	37% (13)	22% (19)	30% (30)	29% (26)	33%[L] (71)

Note: Single response permitted. Mandatory question. Percentages may not sum to 100 due to rounding. Statistically significant differences are calculated at a 95% confidence level and indicated by column letters.

Table 5. Respondents' perceptions of the relationship between GPs and AI in healthcare in the next five years, by gender, age group, role and practice size (% and number)

	Total	Gender		Age group				Role			Practice size by patient number				
	Total (n=1,004)	Male (n=487)	Female (n=497)	35 or under (n=99)	36 to 45 (n=342)	46 to 55 (n=394)	56 or over (n=169)	GP Partner/ Principal (n=438)	Salaried GP (n=395)	Locum GP (n=127)	Up to 5,000 (n=109)	5,001-7,500 (n=155)	7,501-10,000 (n=204)	10,001-12,500 (n=170)	12,501 or more (n=366)
	A	B	C	D	E	F	G	H	I	J	K	L	M	N	O
A simple tool (like a stethoscope)	21% (209)	23%[C] (114)	18% (91)	35%[E,F,G] (35)	23%[G] (79)	18% (71)	14% (24)	26%[I,J] (112)	17% (66)	13% (17)	20% (22)	17% (27)	18% (36)	26% (44)	22% (80)
A helpful collaborator (AI + GP working as a team)	46% (462)	47% (229)	46% (227)	44% (44)	46% (159)	47% (187)	43% (72)	46% (201)	47% (184)	42% (53)	36% (39)	50%[K] (78)	45% (92)	44% (74)	49%[K] (179)
A potential competitor that could replace GP tasks	10% (103)	10% (50)	10% (52)	9% (9)	10% (34)	12% (46)	8% (14)	9% (40)	11% (42)	14% (18)	12% (13)	9% (14)	11% (22)	9% (16)	10% (38)
Unsure	23% (230)	19% (94)	26%[B] (127)	11% (11)	20%[D] (70)	23%[D] (90)	35%[D,E,F] (59)	19% (85)	26%[H] (103)	31%[H] (39)	32%[N,O] (35)	23% (36)	26%[O] (54)	21% (36)	19% (69)

Note: Single response permitted. Mandatory question. Percentages may not sum to 100 due to rounding. Statistically significant differences are calculated at a 95% confidence level and indicated by column letters.

Table 6. Role AI was expected to play in respondents' routine clinical practice in next five years, by gender, age group, role and practice size (% and number)

	**Total**	**Gender**		**Age group**				**Role**			**Practice size by patient number**				
	Total (n=1,004)	Male (n=487)	Female (n=497)	35 or under (n=99)	36 to 45 (n=342)	46 to 55 (n=394)	56 or over (n=169)	GP Partner/ Principal (n=438)	Salaried GP (n=395)	Locum GP (n=127)	Up to 5,000 (n=109)	5,001-7,500 (n=155)	7,501-10,000 (n=204)	10,001-12,500 (n=170)	12,501 or more (n=366)
	A	B	C	D	E	F	G	H	I	J	K	L	M	N	O
Not at all	1% (14)	1% (6)	1% (6)	0% (0)	1% (3)	1%[D] (5)	4%[D] (6)	1% (5)	2% (8)	1% (1)	2% (2)	2% (3)	2% (4)	1% (1)	1% (4)
Minor role	22% (225)	22% (106)	24% (117)	19% (19)	24% (82)	23% (92)	19% (32)	26% (112)	22% (85)	21% (27)	28% (31)	21% (33)	22% (44)	24% (40)	21% (77)
Moderate role	37% (372)	37% (179)	38% (189)	41% (41)	35% (120)	38% (148)	37% (63)	39%[J] (171)	37% (148)	29% (37)	32% (35)	32% (50)	39% (80)	41% (69)	38% (138)
Major role	20% (204)	22% (106)	19% (92)	24% (24)	21% (73)	20% (80)	16% (27)	21% (90)	18% (70)	21% (27)	17% (18)	23% (35)	19% (39)	21% (36)	21% (76)
Transformative	12% (117)	13% (61)	11% (54)	11% (11)	13% (43)	11% (42)	12% (21)	9% (40)	12% (46)	17%[H] (22)	10% (11)	12% (18)	10% (21)	10% (17)	14% (50)
Unsure	7% (72)	6% (29)	8% (39)	4% (4)	6% (21)	7% (27)	12%[D,E] (20)	5% (20)	10%[H] (38)	10%[H] (13)	11%[N] (12)	10%[N] (16)	8% (16)	4% (7)	6% (21)

Note: Single response permitted. Mandatory question. Percentages may not sum to 100 due to rounding. Statistically significant differences are calculated at a 95% confidence level and indicated by column letters.

Table 7. Respondents' views on whether AI will reduce reliance on certain traditional GP skills in the next 10 years, by gender, age group, role and practice size (% and number)

	**Total**	**Gender**		**Age group**				**Role**			**Practice size by patient number**				
	Total (n=1,004)	Male (n=487)	Female (n=497)	35 or under (n=99)	36 to 45 (n=342)	46 to 55 (n=394)	56 or over (n=169)	GP Partner/ Principal (n=438)	Salaried GP (n=395)	Locum GP (n=127)	Up to 5,000 (n=109)	5,001-7,500 (n=155)	7,501-10,000 (n=204)	10,001-12,500 (n=170)	12,501 or more (n=366)
	A	B	C	D	E	F	G	H	I	J	K	L	M	N	O
Yes - AI will replace some core skills	16% (163)	19% (91)	14% (71)	12% (12)	16% (56)	18% (69)	15% (26)	16% (68)	16% (63)	20% (26)	19% (21)	15% (23)	20% (40)	15% (25)	15% (54)
Partly - AI will change which skills matter most	34% (337)	34% (165)	33% (165)	35% (35)	32% (111)	34% (134)	34% (57)	35% (152)	31% (123)	36% (46)	24% (26)	35%[K] (55)	29% (59)	40%[K,M] (68)	35%[K] (129)
No - core GP skills will remain largely unchanged	41% (415	40% (193)	43% (216)	47%[G] (47)	44%[G] (152)	41%[G] (162)	32% (54)	43%[J] (188)	42%[J] (166)	31% (40)	45% (49)	40% (62)	39% (80)	41% (70)	42% (154)
Unsure	9% (89)	8% (38)	9% (45)	5% (5)	7% (23)	7% (29)	19%[D,E,F] (32)	7% (30)	11%[H] (43)	12% (15)	12%[N] (13)	10%[N] (15)	12%[N] (25)	4% (7)	8% (29)

Note: Single response permitted. Mandatory question. Percentages may not sum to 100 due to rounding. Statistically significant differences are calculated at a 95% confidence level and indicated by column letters.

Table 8. Respondents' views on which GP skills or tasks could be most affected by AI in the next 10 years, by gender, age group, role and practice size (% and number)

	Total	Gender		Age group				Role			Practice size by patient number				
	Total (n=915)	Male (n=449)	Female (n=452)	35 or under (n=94)	36 to 45 (n=319)	46 to 55 (n=365)	56 or over (n=137)	GP Partner/ Principal (n=408)	Salaried GP (n=352)	Locum GP (n=112)	Up to 5,000 (n=96)	5,001-7,500 (n=140)	7,501-10,000 (n=179)	10,001-12,500 (n=163)	12,501 or more (n=337)
	A	B	C	D	E	F	G	H	I	J	K	L	M	N	O
Documentation	89% (811)	88% (394)	89% (403)	96%^E,F,G (90)	88% (280)	89% (324)	85% (117)	88% (360)	89% (312)	88% (98)	84% (81)	86% (120)	89% (159)	93%^K,L (152)	89% (299)
Medical knowledge recall	56% (509)	59% (264)	53% (240)	56% (53)	52% (165)	55% (202)	65%^E,F (89)	53% (217)	57% (200)	58% (65)	55% (53)	59%^M (83)	48% (86)	56% (92)	58%^M (195)
Diagnostic problem-solving	48% (442)	55%^C (246)	42% (190)	35% (33)	44% (141)	50%^D (182)	63%^D,E,F (86)	48% (194)	48% (170)	56% (63)	51% (49)	44% (61)	49% (87)	45% (74)	51% (171)
Referral decision-making	42% (383)	48%^C (216)	35% (160)	29% (27)	38% (122)	44%^D (159)	55%^D,E,F (75)	42% (171)	41% (146)	49% (55)	48% (46)	36% (50)	41% (73)	37% (61)	45%^L (153)
Patient communication	35% (319)	35% (158)	35% (157)	33% (31)	35% (111)	31% (114)	46%^D,E,F (63)	34% (139)	34% (120)	42% (47)	28% (27)	37% (52)	35% (63)	37% (60)	35% (117)
Care coordination	32% (291)	34% (152)	30% (137)	31% (29)	26% (83)	34%^E (125)	39%^E (54)	32% (130)	29% (103)	38% (43)	31% (30)	29% (41)	35% (63)	31% (51)	31% (106)
Empathy	6% (53)	5% (22)	7% (30)	4% (4)	5% (16)	7% (24)	7% (9)	5% (21)	6% (21)	7% (8)	5% (5)	8% (11)	7% (13)	5% (8)	5% (16)
Physical examination	4% (37)	4% (20)	4% (17)	2% (2)	3% (11)	5% (18)	4% (6)	4% (16)	3% (12)	7% (8)	4% (4)	6% (8)	4% (8)	4% (7)	3% (10)
Other (please specify)	2% (19)	2% (9)	2% (7)	0% (0)	2%^D (7)	2%^D (8)	3%^D (4)	2% (8)	3% (9)	2% (2)	2% (2)	2% (3)	1% (2)	2% (4)	2% (8)

Note: Selection of multiple options permitted. No limit to number of options selected. Mandatory question. Statistically significant differences are calculated at a 95% confidence level and indicated by column letters.

Table 9. Expectations for AI-based tools on the meaningfulness of GP work in the next 10 years, by gender, age group, role and practice size (% and number)

	Total	Gender		Age group				Role			Practice size by patient number				
	Total (n=1,004)	Male (n=487)	Female (n=497)	35 or under (n=99)	36 to 45 (n=342)	46 to 55 (n=394)	56 or over (n=169)	GP Partner/ Principal (n=438)	Salaried GP (n=395)	Locum GP (n=127)	Up to 5,000 (n=109)	5,001-7,500 (n=155)	7,501-10,000 (n=204)	10,001-12,500 (n=170)	12,501 or more (n=366)
	A	B	C	D	E	F	G	H	I	J	K	L	M	N	O
Make GP work less meaningful	19% (194)	20% (98)	18% (91)	12% (12)	19% (65)	20%^D (79)	22%^G (38)	18% (79)	20% (78)	28%^H (35)	24% (26)	19% (30)	18% (36)	17% (29)	20% (73)

Make GP work more meaningful	22% (225)	28%[C] (134)	18% (89)	25% (25)	25% (84)	21% (81)	21% (35)	23% (102)	21% (81)	17% (22)	18% (20)	20% (31)	24% (49)	21% (36)	24% (89)
Have no impact on the meaningfulness of GP work	37% (367)	34% (165)	39% (196)	42%[G] (42)	37% (128)	38%[G] (148)	29% (49)	40%[J] (174)	37%[J] (148)	25% (32)	36% (39)	37% (58)	36% (73)	36% (61)	37% (136)
Unsure	22% (218)	18% (90)	24%[B] (121)	20% (20)	19% (65)	22% (86)	28%[E] (47)	19% (83)	22% (88)	30%[H] (38)	22% (24)	23% (36)	23% (46)	26% (44)	19% (68)

Note: Single response permitted. Mandatory question. Percentages may not sum to 100 due to rounding. Statistically significant differences are calculated at a 95% confidence level and indicated by column letters.

Table 10. GPs' views on how patients would respond if AI systems took over some routine GP tasks in the next 10 years, by gender, age group, role and practice size (% and number)

	Total	Gender		Age group				Role			Practice size by patient number				
	Total (n=1,004)	Male (n=487)	Female (n=497)	35 or under (n=99)	36 to 45 (n=342)	46 to 55 (n=394)	56 or over (n=169)	GP Partner/ Principal (n=438)	Salaried GP (n=395)	Locum GP (n=127)	Up to 5,000 (n=109)	5,001-7,500 (n=155)	7,501-10,000 (n=204)	10,001-12,500 (n=170)	12,501 or more (n=366)
	A	B	C	D	E	F	G	H	I	J	K	L	M	N	O
Very positively	2% (19)	2% (10)	2% (8)	4% (4)	3% (9)	1% (5)	1% (1)	2% (7)	2% (7)	4% (5)	1% (1)	0% (0)	2%[L] (5)	4%[L] (6)	2%[L] (7)
Mostly positively	15% (154)	20%[C] (97)	11% (57)	18% (18)	15% (50)	15% (59)	16% (27)	17%[J] (76)	14% (57)	10% (13)	13% (14)	20%[M] (31)	11% (22)	17% (29)	16% (58)
Mixed / divided	53% (537)	51% (249)	57% (283)	51% (50)	52% (178)	56% (219)	53% (90)	52% (228)	53% (211)	54% (69)	44% (48)	52% (80)	58%[K] (118)	54% (91)	55% (200)
Mostly negatively	17% (171)	16% (79)	17% (86)	19% (19)	19% (65)	16% (63)	14% (24)	17% (76)	19% (74)	14% (18)	21%[L] (23)	12% (18)	19%[L] (39)	17% (29)	17% (62)
Very negatively	5% (51)	5% (22)	5% (27)	4% (4)	7%[F] (23)	3% (13)	7% (11)	5% (23)	4% (15)	9% (11)	9%[N] (10)	5% (8)	4% (8)	3% (5)	5% (20)
Unsure	7% (72)	6% (30)	7% (36)	4% (4)	5% (17)	9%[D,E] (35)	9% (16)	6% (28)	8% (31)	9% (11)	12%[O] (13)	12%[O] (18)	6% (12)	6% (10)	5% (19)

Note: Single response permitted. Mandatory question. Percentages may not sum to 100 due to rounding. Statistically significant differences are calculated at a 95% confidence level and indicated by column letters.

Table 11. GPs' views on how – if at all – developments in AI tools would affect advice given to someone considering a career as a GP, by gender, age group, role and practice size (% and number)

	Total	Gender		Age group				Role			Practice size by patient number				
	Total (n=1,004)	Male (n=487)	Female (n=497)	35 or under (n=99)	36 to 45 (n=342)	46 to 55 (n=394)	56 or over (n=169)	GP Partner/ Principal (n=438)	Salaried GP (n=395)	Locum GP (n=127)	Up to 5,000 (n=109)	5,001-7,500 (n=155)	7,501-10,000 (n=204)	10,001-12,500 (n=170)	12,501 or more (n=366)

	A	B	C	D	E	F	G	H	I	J	K	L	M	N	O
I would encourage them just as strongly as before	23% (226)	26%[C] (126)	20% (99)	23% (23)	24% (81)	20% (79)	25% (43)	28%[I,J] (121)	19% (75)	13% (17)	20% (22)	16% (25)	21% (43)	23% (39)	27%[L] (97)
I would encourage them, but with reservations	25% (252)	28% (134)	23% (116)	29% (29)	22% (75)	24% (95)	31%[E] (53)	25% (111)	24% (93)	28% (35)	23% (25)	27% (42)	25% (52)	28% (48)	23% (85)
I would be more hesitant than before	14% (137)	13% (62)	14% (71)	9% (9)	14% (49)	15% (61)	11% (18)	13% (57)	14% (56)	17% (22)	17% (18)	17% (26)	12% (25)	14% (24)	12% (44)
I would discourage them	7% (72)	8% (40)	6% (29)	2% (2)	7%[D] (24)	8%[D] (32)	8%[D] (14)	6% (27)	8% (32)	9% (12)	5% (5)	8% (13)	8% (17)	5% (9)	8% (28)
I don't yet feel able to advise either way	32% (317)	26% (125)	37%[B] (182)	36%[G] (36)	33%[G] (113)	32%[G] (127)	24% (41)	28% (122)	35%[H] (139)	32% (41)	36% (39)	32% (49)	33% (67)	29% (50)	31% (112)

Note: Single response permitted. Mandatory question. Percentages may not sum to 100 due to rounding. Statistically significant differences are calculated at a 95% confidence level and indicated by column letters.

Table 12. Expectations on how respondents believe the GP profession will change in the next 20 years, by gender, age group, role and practice size (% and number)

	**Total**	**Gender**		**Age group**				**Role**			**Practice size by patient number**				
	Total (n=1,004)	Male (n=487)	Female (n=497)	35 or under (n=99)	36 to 45 (n=342)	46 to 55 (n=394)	56 or over (n=169)	GP Partner/ Principal (n=438)	Salaried GP (n=395)	Locum GP (n=127)	Up to 5,000 (n=109)	5,001-7,500 (n=155)	7,501-10,000 (n=204)	10,001-12,500 (n=170)	12,501 or more (n=366)
	A	B	C	D	E	F	G	H	I	J	K	L	M	N	O
The GP role will remain largely unchanged	12% (124)	13% (64)	12% (58)	14% (14)	14%[G] (48)	12% (49)	8% (13)	13% (59)	12% (47)	9% (12)	15% (16)	17%[O] (27)	10% (21)	12% (21)	11% (39)
AI will significantly reshape the GP role, but GPs will remain central to care	59% (590)	59% (287)	59% (295)	69%[F] (68)	59% (203)	56% (219)	59% (100)	62%[J] (272)	56% (221)	51% (65)	53% (58)	52% (80)	60% (123)	61% (104)	61%[L] (225)
AI will take over most GP tasks, with	11% (115)	13% (63)	10% (51)	9% (9)	11% (36)	13% (50)	12% (20)	10% (45)	12% (48)	14% (18)	15% (16)	11% (17)	10% (21)	11% (18)	12% (43)

doctors mainly providing oversight															
AI will largely replace GPs, with minimal doctor involvement	3% (31)	3% (14)	3% (17)	0% (0)	2%[D] (8)	4%[D] (15)	5%[D] (8)	2% (8)	3% (11)	9%[H,I] (12)	2% (2)	5% (7)	3% (7)	3% (5)	3% (10)
Unsure	14% (144)	12% (59)	15% (76)	8% (8)	14% (47)	15%[D] (61)	17%[D] (28)	12% (54)	17%[H] (68)	16% (20)	16% (17)	15% (24)	16% (32)	13% (22)	13% (49)

Note: Single response permitted. Mandatory question. Percentages may not sum to 100 due to rounding. Statistically significant differences are calculated at a 95% confidence level and indicated by column letters.
